# The effect of urethroplasty surgery on erectile and orgasmic functions: a prospective study

**DOI:** 10.1590/S1677-5538.IBJU.2018.0276

**Published:** 2019

**Authors:** Ahmet Urkmez, Ozgur H. Yuksel, Emrah Ozsoy, Ramazan Topaktas, Aytac Sahin, Orhan Koca, Metin I. Ozturk

**Affiliations:** 1 Department of Urology, University of Health Sciences, Haydarpasa Numune Research & Training Hospital, Istanbul, Turkey;; 2 Department of Urology, University of Health Sciences, Fatih Sultan Mehmet Research & Training Hospital, Istanbul, Turkey

**Keywords:** Erectile Dysfunction, Prospective Studies, Orgasm

## Abstract

**Objectives:**

to examine the effects of urethroplasty surgery on sexual functions by taking into account age, location of stenosis, length of stenosis and surgical technique parameters.

**Materials and Methods:**

The prospective study was conducted between January 2015 and August 2017 with 60 cases. Patients were categorized according to age groups (19-65 / 65-75 years), surgery technique and stricture localization and length. Before the urethroplasty operation and postoperative 6th month follow-up, the international index of erectile function (IIEF) form (15 questions), was filled, the relevant domains of sexual function; erectile function (Q1,2,3,4,5,15), orgasmic function (Q9,10) and overall satisfaction (Q13,14) were assessed.

**Results:**

The mean age of the cases is 54 ± 13. However, preoperative IIEF, sexual satisfaction and orgasmic function averages of patients with a stenosis segment length of 1-3 cm was found to be significantly higher than that of patients with a stenosis segment length of 4-7 cm. Between stenosis segment length groups, there was no statistical difference in terms of preoperative and postoperative sexual functions. And also, there was no statistically significant change in patients’ preoperative and postoperative sexual function scores in terms of localization of stricture and surgery techniques. However, there were statistically significant change in the postoperative IIEF and sexual satisfaction averages according to preoperative averages.

**Conclusion:**

Our study suggests that urethroplasty surgery itself does not significantly affect erectile function, orgasmic function, and general sexual satisfaction regardless of the type of surgery, localization and length of stenosis. Besides, there was a significant decrease in erectile function in senior adults.

## INTRODUCTION

Urethral stricture refers to the scar formation involving the spongiformis erectile tissue of the corpus spongiosum, resulting in a concomitant narrowing of the urethral lumen. The process leading to this fibrosis is primarily subepithelial inflammation and hemorrhage and later stages are characterized with sclerosis and fibrosis. The etiology of urethral stricture disease is multifactorial and mostly consists of iatrogenic reasons (catheterization / endoscopic procedures) as well as trauma, infections, prostatectomy and other post - prostate cancer treatments, lichen sclerosis and idiopathic reasons. Posterior urethral injuries are often associated with pelvic fractures. Treatment alternatives include; dilatation, internal urethrotomy, laser treatments, and open reconstruction. In the past, known as the reconstructive ladder, it has always been suggested that the simplest procedure should always be tried in the first stage, and in case of failure, proceeding to more complex approaches is recommended in guidelines. In modern urethral reconstruction, this approach is considered outdated. Even in some studies it has been shown that recurrent dilatations and internal urethrotomies reduce the success rate of ultimate open urethral reconstruction (1, 2). In the treatment of urethral strictures, urethroplasty, excision and primary anastomosis are accepted as the “gold standard” treatment method due to low morbidity and cost effectiveness besides long - term high success rates. Certainly there are also strictures that require tissue transfer; grafts and flaps are successfully deployed here (3, 4).

In addition to psychogenic factors, erectile dysfunction (ED) after urethroplasty may be due to damage to the cavernous nerve, damage to the perineal nerve, and deterioration of the flow of the bulbar artery. In order to reduce this damage, it is suggested to protect bulbospongiosus muscle and peroneal nerves, not to cut central tendon, not to damage the bulbar artery, use buccal mucosal graft without cutting corpus spongiosum (5–7). The neurovascular structures responsible for the erectile function pass from a distance of approximately 3 mm from clockwise 1 and 11 right outside the corpus spongiosum and some branches from the cavernous nerves enter the corpus spongiosum across the entire penis. The intercranial area shows that this vessel and nerve bundle is unprotected and vulnerable to trauma. For this reason, the bulb can be very easily damaged during the dissection of the urethra (8–10). Another important point for this region is the damage of the perineal nerve during the separation of the bulbospongiosus muscle on the corpus spongiosum. It provides semen expulsion and sense of penile ventral surface (11). In addition to the effect of the perineal nerve on ejaculation, there is also an effect on erectile function. For this reason, the perineal nerve is thought to be an extra neural pathway for erectile function through unrecognized reflex mechanisms. To protect erectile functions after urethroplasty, it would be beneficial to preserve this neural tissue, if possible (12, 13). In this study, we examined the effects of urethroplasty surgery on sexual functions by taking into account age, location of stenosis, length of stenosis and surgical technique parameters and we hypothesized that location and length of stricture and type of urethroplasty is not affecting sexual functions but older age may affect postoperative sexual functions.

## MATERIAL AND METHODS

Following ethical board approval, the prospective study was conducted between January 2015 and August 2017 with 60 cases aged between 19 and 75 years. Patients under 18 years of age and patients with very poor erectile function (IIEF score ≤ 5) or with no erectile function were excluded. And also, patients with a history of coronary artery bypass graft surgery, unregulated hypertension and diabetes were excluded from the study. Patients were categorized according to age groups (19-65 / 65-75 years) and stricture localization and length. Only six patients with 2 cm stricture at bulbar urethra, had undergone endoscopic intervention once before and failed. All urethroplasty operations were performed by the same surgeon experienced in reconstructive urology. All patients were evaluated with anamnesis, history, physical examination, urine analysis and culture, uroflowmetry, retrograde urethrography before surgery. Before the open urethroplasty operation and postoperative 6th month follow-up, the international index of erectile function (IIEF) form (15 questions) was filled, the relevant domains of sexual function; erectile function (Q1-5, 15), orgasmic function (Q9, 10) and overall satisfaction (Q13, 14) were assessed. The technique of the surgery, localization of the stricture and the length of the narrow segment were also noted.

### Surgical Approach

Urethroplasty was applied for bulbar and penile urethral strictures. This series includes excision and end - to - end anastomoses (using both transecting and non - transecting technique), dorsal onlay buccal mucosal graft, ventral inlay buccal mucosal graft and penile skin flap cases. During operation, surgeon prone to keep the dissection area as small as possible at the bulbar urethral level and did not used cautery during the dissection. And also, surgeon tried to protect bulbospongiosus muscle and peroneal nerves, not to cut central tendon, not to damage the bulbar artery, and used buccal mucosal graft without cutting corpus spongiosum.

### Statistical analysis

While the findings of the study were evaluated, IBM SPSS Statistics 22 program was used for statistical analyzes. When the study data were evaluated, the normal distribution of the parameters was assessed by the Shapiro Wilks test. When study data were evaluated; Kruskal Wallis test was used in comparing quantitative data for comparison of parameters without normal distribution as well as descriptive statistical methods (mean, standard deviation, frequency). Student's t test was used to compare parameters with normal distribution between two groups and Mann Whitney U test was used to compare parameters without normal distribution between two groups. Paired Sample t test was used for intra - group comparison of quantitative data showing normal distribution, Wilcoxon Signed Ranks test was used for intra - group comparison of parameters without normal distribution. Significance was assessed at p < 0.05 level.

## RESULTS

The prospective study was conducted between January 2015 and August 2017 with 60 cases aged between 19 and 75 years. The mean age of the cases was 54.48 ± 13.34. The stricture segment lengths ranged from 2 to 7 cm, with a mean of 3.27 ± 1.04 80% of cases were under 65 years of age, 20% were over 65 years of age. While 75% of the strictures were located at bulbar area, 25% of them were at penile urethra 76.7% had undergone buccal mucosal graft operation, 16.7% had penile skin flap and 6.7% had excision and end - to - end anastomosis operation. In 63.3% of patients, stricture segment length was 3 cm and below; 36.7% of patients had 3 cm and more (Table-1).

**Table 1:** Evaluation of general characteristics of study population.

		Min - Max	Mean ± SD
Age (years)		19-75	54.48 ± 13.34
Length of stenosis segment (cm)		2-7	3.27 ± 1.04
		n	%
**Age (years)**			
	≤ 65	48	80
	> 65	12	20
**Stenosis site**		
	Bulbar	45	75
	Penile Urethra	15	25
**Operation**		
	Buccal mucosal graft	46	76.7
	Penile Skin flap	10	16.7
	End - to - end anastomosis	4	6.7
**Length of stenosis segment**		
	≤ 3 cm	38	63.3
	> 3 cm	22	36.7

Preoperative IIEF values of the patients ranged from 8 to 29, with a mean of 19.93 ± 5.76. Postoperative IIEF values ranged from 6 to 30, with a mean of 20 ± 7.99. There was no statistically significant difference between preoperative and postoperative in terms of IIEF, sexual satisfaction and orgasmic function averages (p > 0.05). When the preoperative and postoperative IIEF scores of the patients are evaluated individually, 27% showed an increase of 4 points or more in the IIEF score, 33% showed a decrease of 4 points or more in the IIEF score, and 40% had no change in the IIEF scores. There was no statistically significant difference in preoperative and postoperative sexual functions of patients who had failed endoscopic procedure once before (p > 0.05).

There was no statistically significant change in patient's preoperative and postoperative IIEF, sexual satisfaction, and orgasmic function averages in terms of localization of stricture, namely being penile or bulbar urethra (p > 0.05) (Table-2).

**Table 2:** Assessment of preoperative and postoperative IIEF, sexual satisfaction and orgasmic function parameters within and between stenosis sites.

		Stenosis site		p^a^
		Bulbar	Penile Urethra	
		Mean ± SD	Mean ± SD	
IIEF	Preoperative	20.87 ± 6.07	21.13 ± 4.93	a10.878
	Postoperative	20.51 ± 7.55	18.47 ± 9.33	a10.396
	Preop - Postoppb1	0.754	0.134	
Sexual satisfaction	Preoperative	10.4 ± 2.27	10 ± 3	a10.589
	Postoperative	10.29 ± 2.34	9.8 ± 3.55	a10.624
	Preop - Postoppb1	0.578	0.595	
Orgasmic function (median)	Preoperative	7.73 ± 1.18 (8)	7.67 ± 0.98 (8)	a20.901
	Postoperative	7.71 ± 1.32 (8)	7.47 ± 1.73 (8)	a20.895
	Preop - Postoppb2	0.888	0.426	

a **=** Comparisons between groups;

b **=** intra - group comparisons.

a1 **=** Student t Test;

a2 **=** Mann Whitney U Test;

b1 **=** Paired Sample's t Test;

b2 **=** Wilcoxon Sign Test;

* = p < 0.05

**IIEF** = International index of erectile function

Preoperative IIEF, sexual satisfaction and orgasmic function averages of patients with a stricture segment length of 1-3 cm was found to be significantly higher than that of patients with a stricture segment length of 4-7 cm (p: 0.004; p: 0.012; p: 0.021). Between stricture segment length groups, there was no statistical difference in terms of preoperative and postoperative IIEF, sexual satisfaction and average orgasmic function (p > 0.05) (Table-3).

**Table 3:** Assessment of preoperative and postoperative IIEF, sexual satisfaction and orgasmic function parameters between and within the stenosis segment length groups.

		Length of stenosis segment		p^a^
		1-3 cm	4-7 cm	
		Mean ± SD	Mean ± SD	
IIEF	Preoperative	22.53 ± 5.18	18.18 ± 5.8	a10.004*
	Postoperative	20.76 ± 7.51	18.68 ± 8.79	a10.335
	Preop - Postoppb1	0.093	0.793	
Sexual satisfaction	Preoperative	10.89 ± 2.18	9.27 ± 2.6	a10.012*
	Postoperative	10.66 ± 2.36	9.32 ± 3	a10.060
	Preop - Postoppb1	0.230	0.870	
Orgasmic function (median)	Preoperative	7.97 ± 1.1 (8)	7.27 ± 1.03 (7)	a20.021*
	Postoperative	7.89 ± 1.37 (8)	7.23 ± 1.45 (7.5)	a20.083
	Preop - Postoppb2	0.570	0.858	

a = Comparisons between groups;

b = intra - group comparisons.

a1 **=** Student t Test;

a2 = Mann Whitney U Test;

b1 **=** Paired Sample's t Test;

b2 **=** Wilcoxon Sign Test;

* = p < 0.05

**IIEF =** International index of erectile function

According to type of operations, namely Buccal mucosal graft, Penile skin flap and end - to - end anastomosis operation, there was no statistically significant difference in terms of preoperative and postoperative IIEF, sexual satisfaction and orgasmic function averages of the patients (p > 0.05) (Table-4).

**Table 4:** Assessing preoperative and postoperative IIEF, sexual satisfaction and orgasmic function parameters between and within surgery types.

Surgery	IIEF		Preop - Postop p^2^
	Preoperative	Postoperative	
	Mean ± SD (median)	Mean ± SD (median)	
Buccal mucosal graft	20.91 ± 5.7 (21)	20.67 ± 7.21 (21)	0.807
Penile Skin flap	19.1 ± 6.1 (19.5)	16.2 ± 11.01 (14)	0.183
End - to - end anastomosis	25.75 ± 3.59 (26.5)	21.75 ± 7.27 (21.5)	0.285
p^1^	0.159	0.533	
	**Sexual satisfaction**		
	Preoperative	Postoperative	Preop - Postop p^2^
	Mean ± SD (median)	Mean ± SD (median)
Buccal mucosal graft	10.28 ± 2.18 (10)	10.26 ± 2.29 (10)	0.872
Penile Skin flap	9.8 ± 3.39 (10.5)	9.5 ± 4.25 (9.5)	0.457
End - to - end anastomosis	11.75 ± 2.99 (12)	10.75 ± 2.22 (11)	0.180
p^1^	0.572	0.873	
	**Orgasmic function (median)**		
	Preoperative	Postoperative	Preop - Postop p^2^
	Mean ± SD (median)	Mean ± SD (median)
Buccal mucosal graft	7.78 ± 1.07 (8)	7.83 ± 1.16 (8)	0.820
Penile Skin flap	7.2 ± 1.23 (7.5)	6.8 ± 2.15 (7.5)	0.271
End - to - end anastomosis	8.25 ± 1.26 (8)	7.75 ± 1.71 (7.5)	0.157
p^1^	0.343	0.496	

1 = Kruskal Wallis Test;

2 = Wilcoxon Sign Test;

IIEF = International index of erectile function

According to age groups, preoperative sexual satisfaction averages of those aged 65 and below were statistically significantly higher than those of older than 65 years (p: 0.000). However, there were no statistically significant difference in the mean preoperative IIEF and orgasmic function (p: 0.057; p: 0.070). Again, postoperative IIEF, sexual satisfaction and orgasmic function averages of those aged 65 and below were found to be statistically significantly higher than those of those older than 65 years (p: 0.002; p: 0.000; p: 0.004).

In patients younger than 65 years, there was no statistically significant change in the postoperative IIEF, sexual satisfaction, and orgasmic function averages according to preoperative averages (p > 0.05). In patients older than 65 years of age, there was no statistically significant change in the postoperative orgasmic function averages (p > 0.05). However, there were statistically significant change in the postoperative IIEF and sexual satisfaction averages according to preoperative averages (p: 0.020; p: 0.021) (Table-5).

**Table 5:** Assessment of preoperative and postoperative levels of IIEF, sexual satisfaction and orgasmic function parameters among and between age groups.

		Age		p^a^
		≤ 65	> 65	
		Mean ± SD	Mean ± SD	
IIEF (median)	Preoperative	21.68 ± 5.41 (22)	17.91 ± 6.34 (18)	a20.057
	Postoperative	21.60 ± 7.17 (22.5)	13.58 ± 8.17 (12)	a20.002*
	Preop - Postoppb2	0.877	0.020*	
Sexual satisfaction	Preoperative	11.02 ± 1.92	7.41 ± 223	a10.000*
	Postoperative	11.04 ± 2.05	6.66 ± 1.87	a10.000*
	Preop - Postoppb1	0.908	0.021*	
Orgasmic function (median)	Preoperative	7.85 ± 1.03 (8)	7.16 ± 1.33 (7)	a20.070
	Postoperative	7.91 ± 1.25 (8)	6.58 ± 1.62 (7)	a20.004*
	Preop - Postoppb2	0.719	0.053	

a = Comparisons between groups;

b = intra - group comparisons

a1 = Student t Test;

a2 = Mann Whitney U Test;

b1 = Paired Sample's t Test;

b2 = Wilcoxon Sign Test;

* = p < 0.05

IIEF = International index of erectile function

## DISCUSSION

Sexual dysfunction after urethroplasty is a very broad definition that also includes disorders of erectile dysfunction, ejaculatory disorders, penile curvature or chordee and genital sensitivity disorders. Current literature shows that sexual dysfunction after urethroplasty is not very common, approximately 1% (0% – 38%) after anterior urethroplasty and approximately 3% (0% – 34%) after pelvic fracture related urethral injury repair (6).

In some studies it was thought that the age of the patient, pre - surgical sexual function, previous surgical intervention, post - operative survival, length of stenosis and severity of stricture might be factors affecting long - term erectile function after urethroplasty (14–16). The first article on continuous ED development after urethroplasty was written by Mundy et al. (17). In their series including 200 patients, permanent ED rate was reported as 5% after anastomotic urethroplasty and 0.9% after graft urethroplasty.

The relationship between ED after the urethroplasty and the patient's age was different in different studies. A study by Johannes and his colleagues found that the ED frequency decreased as the age of the patients decreased. When the patient's age was around 40, ED frequency was 5% and when the patient's age was around 70, ED frequency was 15% (16). A retrospective study showed that ED, with a frequency of 25% after urethroplasty, was rare in 1 year after surgery and in young men (14). In another study, it was shown that patient age is important in improving ED after urethroplasty. It was stated that the time required for complete recovery of erectile function after surgery in patients under 40 years is 6 months (18). In a prospective study conducted by Erickson et al., anterior urethroplasty resulted in ED in approximately 40% of patients, with healing occurring at most in the first 6 months. They stated that bulbar urethroplasty can affect erectile function more than penile urethroplasty and this can be explained with the fact that bulbar urethra is located closer to the nerve responsible for the erection (11). Haines et al. found that being older than 50 years was associated with a decrease in postoperative erectile function. This relationship has also been explained by reduced tissue plasticity, poor recovery, or perhaps even more co - morbidity of the elderly cohort, and thus greater susceptibility to ED development (19). There are studies that show that age has a little effect on urinary and sexual functions after urethroplasty as opposed to these (20). In our study, patients were evaluated in two different groups including patients younger than 65 years of age and patients older than 65 years of age. In patients younger than 65 years of age, there was no statistically significant difference in terms of preoperative and postoperative sexual functions. However, in patients older than 65 years of age, there were statistically significant difference in the postoperative IIEF and sexual satisfaction averages according to preoperative averages. In addition, while there was no significant difference between age groups in terms of preoperative IIEF and orgasmic functions, there was a statistically significant difference between the two groups in the postoperative averages. Although we cannot make an optimal assessment because of the small number of patients in this group of older than 65 years, these results suggest that the senior adult group may be more affected by urethroplasty surgery in terms of sexual functions.

When studies were examined, the type of urethroplasty applied was thought to be effective in the formation of ED. In a study involving eighty - nine patients, patients were divided into 3 groups according to urethroplasty. Penile substitution urethroplasty, bulbar excision - anastomosis and bulbar substitution urethroplasty were compared. Average follow-up time was 15 months. Stricture length and patient age were statistically similar in all groups. The authors noted that the type of urethroplasty applied did not have a significant effect on the development of ED and that erectile function after urethroplasty was improved within the first 6 months. The same authors also recommended early use of phosphodiesterase type – 5 inhibitors and nonsteroidal anti-inflammatory drugs (21). In the literature review of Dogra et al., they reported that in anterior urethroplasty, transecting bulbar urethroplasty has a higher risk of developing sexual dysfunction compared to non - transecting or penile urethroplasty (22). Along with similar studies in the literature, Haines et al., conducted a prospective study with 87 patients and found no relation between urethral transecting and increased erectile dysfunction, and detected that the localization of stricture had no effect on this situation (19). In our study, there was no statistically significant difference for preoperative and postoperative IIEF scores, orgasmic function and sexual satisfaction according to stenosis localization and surgery type.

Initially, de novo ED after urethroplasty was shown to be higher in elderly patients and those with long stricture segments (6.8 cm vs. 4.4 cm) (23). Further work, however, has disproved this relationship. And in a recent meta - analysis, no relationship was found between the length of stricture and the incidence of postoperative ED (6, 24). According to Coursey et al., the more frequent drop in erectile function seen in patients treated with penile skin flap has been attributed to the temporary change in penile external appearance in these patients and the long stricture segments (23). In our study, although the preoperative erectile function of the patients with the long stricture segment was lower than the patients with the short stricture segment (1-3 cm), there was no significant change in the erectile function of these patients in the postoperative period.

In another study, most patients complained of sexual dysfunction, especially about reduced ejaculatory fluid (85%) before urethroplasty and after urethroplasty, no one reported a worsening erection; and many of them reported that there was a significant improvement in erection, ejaculation, relationship with their partner, sexual activity and desire (25). Erickson et al., have found that urethral reconstructive surgery affects ejaculatory functions positively while not significantly affecting erectile function or sexual dysfunction. However, older men reported that they had more ED after the surgery than younger men, but that erectile function probably improved over time (14). In our study, in terms of preoperative IIEF, orgasmic function and sexual satisfaction, there were statistically significant differences between the stricture length groups of 1-3 cm and 4-7 cm. Also, there were significant differences both preoperative and postoperative scores between the age groups of below and above 50.

The length of the urethral stricture is closely related to the grade of fibrosis in the urethra and surrounding tissues. Among the causes of long urethral strictures it is included inflammatory diseases, recurrent urethral dilatations, long - term urethral catheterizations, and traumatic urethral injuries. In the literature review of Palminteri et al. (5), they reported that factors such as the initial sexual function of the patient, the etiologic agent causing the urethral stricture, the psychological factors associated with the stenosis or the duration of the treatment, postoperative tissue edema or inflammation or urethroplasty surgery, might cause ED.

Our study includes some limitations although it has a prospective design. A single surgeon in a single center conducted all of the urethroplasties. But it is acceptable to consider that surgical techniques and stricture etiology may significantly affect to the outcomes. Consequently, this may limit the generalizability of our results. Our study population of 60 patients may be lacking to detect small changes in erectile functions that may occur after urethroplasty. Larger patient numbers and multicentric studies will provide more accurate and generalized results. In addition, limitations of our study include the lack of patient's preoperative hormonal and andrological evaluation and use of objective parameters such as semen volume and the absence of validated questionnaires (MSHQ and BMFSI) for assessment of orgasmic - ejaculatory functions.

## CONCLUSION

Our study suggests that urethroplasty surgery itself does not significantly affect erectile function, orgasmic function, and general sexual satisfaction regardless of the type of surgery, localization and length of stricture. Besides, there was a significant decrease in erectile function in senior adults. Other factors such as hypertension and vascular disease with older age may play a role in decrease in erectile function following urethroplasty. Although patients with long stricture segments have lower preoperative sexual function scores, urethroplasty surgery does not significantly affect postoperative sexual outcome in experienced centers. We think that in endoscopic treatment resistant short segment strictures and long segment strictures, urethroplasty is the gold standard treatment option that can be safely used in terms of surgical success and sexual function parameters.

## ETHICAL APPROVAL

All procedures performed in studies involving human participants were in accordance with the ethical standards of the institutional and / or national research committee and with the 1964 Helsinki declaration and its later amendments or comparable ethical standards.

Informed consent: Informed consent was obtained from all individual participants included in the study.
